# Can the Cognitive Function Index identify cognitive impairment in Latin America?

**DOI:** 10.1002/alz70857_103411

**Published:** 2025-12-25

**Authors:** Monica Sanches Yassuda, Luc¡a Crivelli, Claudia Kimie Suemoto, Ismael Luis Calandri, Rosa Maria Salinas‐Contreras, Natalia Acosta‐Baena, Paulo Caramelli, Sonia Maria Dozzi Brucki, Gustavo Henrique Cançado, Ana Vigil‐Martinez, Maria Eugenia Martin, Ezequiel Ignacio Surace, Maria Da Graça Morais Martin, Andrés Damian, Belen Custodio, Nicolas Corvalan, Diego Fernandez Slezak, Lina Marcela Vellila, Luzia Lima Carreira, Adalberto Studart Neto, Eduardo Sturzeneker Tres, Leonel Tadao Takada, Karolina G Cesar, Jerusa Smid, Silvia Stahl Merlin, Valquíria de Oliveira Bárbara, Isabella B Avolio, Maria Aquimara Zambone Magalhães, Cristiane Peixoto, Clarisse Vasconcelos Friedlaender, Daisy M Acosta, David Fernando Aguillón Niño, Ana Charamelo, Nilton Custodio, Carolina Delgado, María Isabel Cusicanqui, Jorge M Leon‐Salas, Lissette Duque, Ivonne Z. Jimenez Velazquez, Heather M Snyder, Maria C. Carrillo, Miia Kivipelto, Ana Luisa Sosa, Gustavo Sevlever, Ricardo Nitrini, Ricardo Allegri

**Affiliations:** ^1^ Universidade de São Paulo, São Paulo, São Paulo, Brazil; ^2^ Fleni, Buenos Aires, Argentina; ^3^ Division of Geriatrics, Department of Internal Medicine, University of São Paulo Medical School, São Paulo, São Paulo, Brazil; ^4^ Fleni, Buenos Aires, Buenos Aires, Argentina; ^5^ National Instutute of Neurology and Neurosurgery, Mexico City, Mexico; ^6^ Grupo de Neurociencias de Antioquia, Facultad de Medicina, Universidad de Antioquia, Medellín, Antioquia, Colombia; ^7^ Universidade Federal de Minas Gerais, Belo Horizonte, MG, Brazil; ^8^ University of São Paulo Medical School, São Paulo, Brazil; ^9^ Universidade Federal de Minas Gerais, Belo Horizonte, Minas Gerais, Brazil; ^10^ Instituto Nacional de Ciencias Médicas y Nutrición Salvador Zubirán, Mexico City, QR, Mexico; ^11^ LIM44, Hospital das Clinicas HCFMUSP, Faculdade de Medicina, Universidade de Sao Paulo, Sao Paulo, Sao Paulo, Brazil; ^12^ Centro Uruguayo de Imagenología Molecular, CUDIM., Montevideo, Montevideo, Uruguay; ^13^ Unit Cognitive Impairment and Dementia Prevention, Peruvian Institute of Neurosciences, Lima, Peru, Lima, Lima, Peru; ^14^ University of Buenos Aires, Ciudad Autonoma de Buenos Aires, CABA, Argentina; ^15^ Grupo de Neurociencias de Antioquia, Universidad de Antioquia, Medellin, Colombia; ^16^ LIM44, Departamento de Radiologia e Oncologia, Faculdade de Medicina da Universidade de São Paulo, Sao Paulo, Brazil; ^17^ Cognitive and Behavioral Neurology Unit, Hospital das Clinicas HCFMUSP, Faculdade de Medicina, Universidade de Sao Paulo, Sao Paulo, Sao Paulo, Brazil; ^18^ University of São Paulo, São Paulo, Brazil; ^19^ University of São Paulo Medical School, Sao Paulo, Brazil; ^20^ Cognitive and Behavioral Neurology Unit ‐ University of São Paulo, São Paulo, Brazil; ^21^ University of São Paulo, São Paulo, São Paulo, Brazil; ^22^ Universidad Nacional Pedro Henriquez Ureña, Santo Domingo, Distrito Nacional, Dominican Republic; ^23^ Grupo de Neurociencias de Antioquia, Universidad de Antioquia, Medellín, Antioquia, Colombia; ^24^ Centro Uruguayo de Imagenología Molecular ‐ CUDIM, Montevideo, Uruguay; ^25^ Research unit, Instituto Peruano de Neurociencias, Lima, Peru; ^26^ Universidad de Chile, Santiago, Santiago, Chile; ^27^ Centro Neurológico Mente Activa, La Paz, Bolivia (Plurinational State of); ^28^ Clínica Bíblica Hospital, San José, Costa Rica; ^29^ Neuromedicenter, Quito, Ecuador; ^30^ University of Puerto Rico, School of Medicine, San Juan, Puerto Rico, PR, USA; ^31^ Alzheimer's Association, Chicago, IL, USA; ^32^ Department of Neurology, Institute of Clinical Medicine, University of Eastern Finland, Kuopio, Finland; ^33^ Instituto Nacional de Neurología y Neurocirugía, Mexico City, DF, Mexico; ^34^ Fleni, CABA, Buenos Aires, Argentina; ^35^ Universidade de Sao Paulo, Sao Paulo, SP, Brazil

## Abstract

**Background:**

The Cognitive Function Index (CFI) was developed to identify individuals with Subjective Cognitive Decline (SCD), which is defined by the self‐perception of cognitive impairment, not detected in neuropsychological tests. Limited research has been done with this tool in Latin America (LA). We aimed to investigate the association between CFI and global cognition and to investigate whether CFI scores can identify participants with cognitive performance below 1.0 standard deviation (SD) of the sample mean.

**Method:**

The LatAm‐FINGERS study is a dementia prevention feasibility study in 12 LA countries. For the present analysis, 815 participants with complete cognitive data (604 women, 74.1%; 436 mestizo 53.5%; mean age=67.5, SD=4.8; mean education = 13.0, SD=3.6; mean GDS=2.7, SD=2.8) were included. The CFI is a 14‐item self‐report measure of cognitive change; higher scores indicate higher concerns. CFI total score was normalized into a z‐score, as some questions were not applicable to all participants and total score differed among them. Cognition was assessed with the Preclinical Alzheimer Cognitive Composite (PACC‐5) ‐ Mini‐Mental State Examination; Logical Memory Delayed Recall; Free and Cued Selective Reminding Test; Digit Symbol Substitution Test; and Animal Category Fluency. Participants were classified into those with PACC‐5 at or above 1 SD from the mean (*n* = 764) and those below (*n* = 51). Bivariate and partial correlations (controlling for sex, age, education and GDS score) were used to assess the association between the CFI and PACC‐5 scores. Receiver operating characteristic (ROC) analyses were used to assess CFI accuracy to identify participants with PACC‐5 scores below 1 SD.

**Result:**

CFI and PACC‐5 scores were negatively associated with each other (*r* =  ‐0.27; *p* <0.001; partial r = ‐0.12; *p* <0.001). ROC analyses indicated an area under the curve (AUC) of 0.71 (sensitivity=0.64; specificity=0.70) with an optimal threshold of 0.36 (Youden Index=0.35) as a suggested cutoff score for the normalized CFI score.

**Conclusion:**

The CFI may be added to cognitive screening protocols to identify individuals who may need further assessment. Integrating the CFI into screening strategies in LA could play a pivotal role in reducing cognitive health disparities and advancing tailored dementia prevention initiatives that address the region's specific sociocultural and healthcare challenges.

